# Transumbilical laparoscopic-assisted appendectomy: a feasible minimally invasive option for pediatric acute appendicitis with comparable outcomes to three-port laparoscopic appendectomy

**DOI:** 10.3389/fped.2026.1724445

**Published:** 2026-05-01

**Authors:** Yifan Li

**Affiliations:** Department of Pediatric Surgery, Maternity & Child Healthcare Hospital of Longgang District, Shenzhen City (Longgang Maternity and Child Institute of Shantou University Medical College), Shenzhen, China

**Keywords:** acute appendicitis, children, laparoscopic appendectomy, minimally invasive surgery, transumbilical laparoscopic-assisted appendectomy

## Abstract

**Objective:**

This study aimed to compare the safety and feasibility of transumbilical laparoscopic-assisted appendectomy (TULAA) vs. three-port laparoscopic appendectomy (TPLA) for the treatment of acute appendicitis in children.

**Materials and methods:**

A retrospective analysis was conducted on pediatric patients who underwent appendectomy at Maternity&Child Healthcare Hospital of Longgang District, Shenzhen City (Longgang Maternity and Child Institute of Shantou University Medical College) between January 2022 and June 2025. Patient demographics, operative times, postoperative recovery parameters (time to first flatus, length of hospital stay), and complication rates were compared between the two surgical groups.

**Results:**

A total of 171 patients were enrolled in the study, with 109 cases in the TULAA group and 62 cases in the TPLA group. There were no significant differences between the TULAA and TPLA groups in baseline characteristics including age, gender, weight, height, BMI, white blood cell count, or CRP levels (*p* > 0.05). The mean operative time was comparable between groups (TULAA: 54.94 ± 24.97 min vs. TPLA: 58.65 ± 25.58 min, *p* = 0.356). However, the TULAA group demonstrated a significantly shorter average hospital stay (4.83 ± 1.53 days vs. 5.35 ± 1.19 days, *p* = 0.022). Conversely, the time to first postoperative flatus was significantly longer in the TULAA group (25.81 ± 11.00 h vs. 21.13 ± 10.01 h, *p* = 0.006). The overall complication rate showed no significant difference (TULAA: 4.6% vs. TPLA: 6.5%, *p* > 0.05), with all cases managed conservatively.

**Conclusion:**

For pediatric acute appendicitis, TULAA is a safe and feasible minimally invasive technique, resulting in a significantly reduced hospital stay compared to TPLA despite a slightly delayed return of bowel function. Both techniques demonstrate comparable operative times and overall complication rates. TULAA represents a valuable surgical option within the pediatric minimally invasive arsenal.

## 1 Introduction

Acute appendicitis represents a common and significant pediatric surgical emergency, with a lifetime incidence of approximately 7%–8% (1, 2). Annually, a significant number of children worldwide require surgical intervention as a result of acute appendicitis (3).

At present, there is no unified conclusion on the causes of appendicitis, and existing research tends to be related to appendiceal duct obstruction, lymphoid tissue hyperplasia, and other factors. In addition, some studies suggest that appendicitis may have a genetic predisposition, with some patients with a positive family history having a higher incidence of appendicitis.

The typical symptom of acute appendicitis in children is metastatic abdominal pain, which moves from periumbilical pain to the lower right abdomen, but there are also some children who only present with periumbilical pain. In addition, there are some other symptoms such as decreased appetite, nausea and vomiting, fever, etc. At present, the common laboratory tests for diagnosing appendicitis in China are white blood cell count (WBC), absolute neutrophil count (ANC), and C-reactive protein (CRP). The preferred imaging method for diagnosing appendicitis in children is color Doppler ultrasound examination.

The management of appendicitis in children has evolved considerably over time, transitioning from primarily open surgical techniques to a greater emphasis on minimally invasive approaches (4). Traditionally, open appendectomy, involving a direct incision to access and remove the appendix, was the standard surgical approach. While effective, open appendectomy is associated with larger incisions, potentially increased postoperative pain, longer recovery times, and a higher risk of wound complications compared to minimally invasive techniques.

The feasibility and safety of TPLA has been verified (5, 6). In recent years, TULAA has emerged as a further refinement of the minimally invasive approach to appendectomy in children (4). The safety and feasibility of TULAA in the treatment of emergency appendicitis, especially its application value for pediatric patients, remain unclear at present.

This study aims to compare the safety and feasibility of TULAA and TPLA in the treatment of acute appendicitis in children.

## 2 Methods

### 2.1 Study population and design

We conducted a retrospective study that included pediatric patients with appendicitis who received TPLA and TULAA at Maternity&Child Healthcare Hospital of Longgang District, Shenzhen City (Longgang Maternity and Child Institute of Shantou University Medical College) from January 2022 to June 2025. This study was approved by the Ethics Committee of Maternity&Child Healthcare Hospital of Longgang District, Shenzhen City (Longgang Maternity and Child Institute of Shantou University Medical College). All pediatric patients have obtained written informed consent from their parents/legal guardians before undergoing surgery. All treatments strictly followed relevant guidelines and regulations.

The exclusion criteria were: appendiceal tumor or unclear pathological diagnosis; If other parts were found during the operation, surgery should be performed simultaneously (such as inguinal hernia, Meckel's diverticulum, etc.); and incomplete clinical data.

From January 2022 to June 2023, our center performed TPLA exclusively for children with appendicitis. Since July 2023, TULAA has been implemented at our center and has become the preferred surgical treatment option for children with appendicitis; TPLA is used only when the patient's parents refuse TULAA. All laparoscopic surgeries were performed by two experienced surgeons. A retrospective analysis of the cases was conducted, with statistical data including patients' demographic characteristics, clinical manifestations, surgical outcomes, complications, and pathological examination findings. The diagnosis of appendicitis was confirmed based on the pathological examination results.

Distribution test of continuous variables: Before statistical analysis, the normality of continuous variables (e.g., operative time, length of hospital stay, time to first flatus) was tested using the Shapiro–Wilk test. Variables with a normal distribution (*p* > 0.05) were presented as mean ± standard deviation (x¯ ± s) and compared using independent samples t-test; variables with a skewed distribution (*p* < 0.05) were presented as median (interquartile range) and compared using the Mann–Whitney U test.

Definition of key statistical indicators: (1) Complication rate:number of patients with postoperative complications/total number of included patients×100%, including wound infection and intra-abdominal abscess within 30 days after surgery; (2) Length of hospital stay was calculated from the day of surgery to the day of discharge (excluding cases of prolonged hospitalization due to non-surgical reasons such as upper respiratory tract infection); (3) Time to first flatus was recorded as the objective time documented by nurses after patient reporting.

Handling of categorical variables: Categorical variables (e.g., gender, complication type) were presented as *n* (%), and compared using the chi-square test. When the expected frequency of any cell was < 5, Fisher's exact test was used instead.

Statistical software and parameters: All statistical analyses were performed using SPSS 20.0 (SPSS Inc., Chicago, IL, USA) and R 4.2.0 (packages: “nortest” for normality test, "stats" for group comparison). A two-tailed *p* < 0.05 was considered statistically significant, and no multiple comparison correction was needed as the study focused on pre-specified primary and secondary outcomes without exploratory analysis.

### 2.2 Description of technique

#### 2.2.1 TULAA: transumbilical laparoscopic assisted appendectomy

A 10-mm incision was made below the umbilicus, and a 7 mmHg pneumoperitoneum was established using a disposable single-port trocar device. Under laparoscopic assistance, the appendix was identified. Adhesions between the appendix and surrounding tissues were lysed if necessary. The appendix was then exteriorized through the umbilical incision and resected extracorporeally. Pneumoperitoneum was re-established, and the abdominal cavity was irrigated if needed. The incision was closed with absorbable sutures, and the skin was apposed with medical adhesive.

#### 2.2.2 TPLA: three-port laparoscopic appendectomy

A 10-mm trocar was inserted through the subumbilical incision, and two additional 5-mm operative trocars were placed in the left lumbar region and the suprapubic area. The appendix was resected intra-abdominally and then extracted through the umbilical incision.

## 3 Results

### 3.1 Study population enrollment

From March 2022 to July 2025, a total of 171 children with appendicitis underwent surgical treatment at our center. Among them, 109 children (63.7%) were treated with TULAA, and 62 children (36.3%) were treated with TPLA. None of the laparoscopic surgery cases in this study were converted to open surgery. The demographic characteristics of the children are detailed in Table 1.

**Table 1:** Demographic and clinical characteristics of pediatric patients diagnosed with appendicitis and treated with TULAA or TPLA, *N* = 171.

Variable	TULAA *N* = 109	TPLA *N* = 62	*P*
Age (years)	8.9 ± 3.2	8.7 ± 2.8	0.692
Gender, male	61	41	0.200
Weight, kg	31.4 ± 12.6	30.4 ± 12.2	0.610
Height, cm	134.9 ± 19.6	132.4 ± 18.3	0.631
BMI, kg/m^2^	16.8 ± 2.9	15.6 ± 2.9	0.590
White blood cell preoperative	12.7 ± 6.5	14.7 ± 6.3	0.052
C-reactive protein preoperative	22.2 ± 38.5	27.7 ± 42.0	0.386
Complicated appendicitis	15	11	0.512
OR time, minutes	54.9 ± 25.0	58.7 ± 25.6	0.356
Complications (any)	5	4	0.725
The time to first postoperative flatus,hours	25.8 ± 11.0	21.1 ± 10.0	0.006
Hospital stay, days	4.8 ± 1.5	5.4 ± 1.2	0.022

### 3.2 Baseline demographic characteristics

There was no significant difference in age (*p* = 0.692) and gender distribution (*p* = 0.200) between the TPLA group and the TULAA group (*p* = 0.692). Additionally, no significant differences were observed between the two groups in terms of weight, height, body mass index (BMI), preoperative white blood cell count, and preoperative C-reactive protein (CRP) levels. Only two patients were classified as overweight or obese.

### 3.3 Perioperative surgical outcomes

The operative time in the TULAA group was 54.9 ± 25.0 min, and in the TPLA group was 58.7 ± 25.6 min, with no statistically significant difference between the two groups (*p* = 0.356). However, the average hospital stay for children treated with TULAA was 0.52 days shorter than that in the TPLA group (4.8 ± 1.5 days vs. 5.4 ± 1.2 days, *p* = 0.022).Notably, the time to first postoperative flatus in the TULAA group was significantly longer than that in the TPLA group, with an average prolongation of 4.7 h (25.8 ± 11.0 h vs. 21.1 ± 10.0 h, *p* = 0.006).

### 3.4 Postoperative complication rates

In the TULAA group, 5 children developed postoperative complications, including 3 cases of wound infection and two cases of intra-abdominal abscess. In the TPLA group, 4 children experienced complications, consisting of two cases of wound infection and two cases of intra-abdominal abscess. There was no significant difference in the overall complication rate between the two groups. All 9 children recovered with conservative treatment and were subsequently discharged.

### 3.5 Outcomes in complicated appendicitis subgroup

We also analyzed the data of cases with complicated appendicitis. As shown in Table 2, for cases of complicated appendicitis, regardless of whether TPLA or TULAA was performed, there were no significant differences between the two groups in terms of operative time, hospital stay, and time to first postoperative flatus.

**Table 2:** Clinical characteristics of pediatric patients diagnosed with complicated appendicitis and treated with TULAA or TPLA, *N* = 26.

Variable	TULAA *N* = 15	TPLA *N* = 11	*P*
OR time, minutes	85.7 ± 37.7	84.7 ± 26.8	0.944
The time to first postoperative flatus, hours	33.7 ± 12.3	10.3 ± 3.1	0.115
Hospital stay, days	7.4 ± 2.4	6.9 ± 1.0	0.526

## 4 Discussion

Uncomplicated acute appendicitis in children can be treated either non-surgically (with antibiotics only) or surgically (appendectomy). In surgical treatment, laparoscopic surgery is the preferred option. Complicated appendicitis is defined as cases where there is a visible perforation of the appendix, fecaliths in the abdominal cavity, or the formation of an abscess. The complication rate of surgical treatment for appendicitis is between 5% and 15%, including abdominal abscesses, incision infections, and intestinal obstruction.

Acute appendicitis represents a common and significant pediatric surgical emergency, demanding prompt diagnosis and intervention to minimize morbidity. The management of appendicitis in children has evolved considerably over time, transitioning from primarily open surgical techniques to a greater emphasis on minimally invasive approaches. While TPLA remains a standard pediatric appendectomy procedure with established safety and efficacy, TULAA is gaining popularity as a safe and effective alternative in selected pediatric cases.

TULAA combines the benefits of laparoscopy with a single umbilical incision. The technique involves using laparoscopic visualization to identify and mobilize the appendix, followed by its exteriorization through the umbilicus for extracorporeal ligation and resection (7, 8). This approach aims to further minimize scarring, potentially improve cosmetic outcomes, and reduce postoperative pain. Some studies suggest TULAA can lead to shorter operative times and hospital stays, and lower costs compared to conventional laparoscopic appendectomy (4, 7, 9). A retrospective study found that simple ligation does not compromise treatment efficacy, increase postoperative complications, or prolong hospital stay (10).

The simplicity of TULAA also contributes to its appeal. The procedure is considered easy to learn and fast to perform. The reduced operative time compared to TPLA have been demonstrated in several studies, further enhancing its efficiency. By minimizing the need for specialized equipment and shortening operative times, TULAA presents a resource-sensitive alternative without sacrificing patient safety.

Numerous studies indicate that TULAA is associated with significantly shorter operative times compared to LA for uncomplicated appendicitis. A meta-analysis of 16 studies including 5,084 pediatric patients showed that TULAA had a significantly shorter operative time compared to TPLA (4). This finding is further supported by single-center studies. For instance, one study involving 1,154 patients found that TULAA reduced operative time by an average of 5.2 min (11). Similarly, another study reported a mean operative time of 30.39 min for TULAA, significantly shorter than the 47.83 min for TPLA (12). A retrospective review reported a mean operative time of just 21 min for TULAA cases, compared to 37 min for TPLA cases (7).

In addition to shorter operative times, TULAA has also been associated with reduced hospital stays for pediatric patients with uncomplicated appendicitis. The meta-analysis mentioned above confirmed that TULAA was associated with a reduced hospital stay compared to TPLA in uncomplicated cases (4). This is consistent with the results of several individual studies. One study reported that patients undergoing TULAA were more likely to be discharged within 24 h (11). Another study found a significantly shorter length of stay for the TULAA group (2.54 days) compared to the TPLA group (3.22 days) (12). Similarly, research showed that the mean hospital stay for TULAA cases was 1.6 days, vs. 2.4 days for TPLA cases (7).

Earlier ambulation and oral intake postoperatively contribute to faster recovery and shorter hospital stays. One study specifically highlighted that the first day of oral intake after surgery was earlier in the TULAA group (1.05 days) compared to the TPLA group (1.32 days) (12).

In summary, evidence suggests that TULAA offers advantages over TPLA in terms of operative time and length of hospital stay for uncomplicated pediatric appendicitis. These benefits are further supported by earlier ambulation and oral intake post-surgery.

Multiple studies have compared the incidence of postoperative complications between TULAA and TPLA. A meta-analysis of 16 studies, encompassing 5,084 pediatric patients, revealed that no significant differences in wound infection rates, ileus, readmission rates, or conversion rates between the two approaches (4).

While TULAA has demonstrated efficacy in managing pediatric appendicitis, particularly in uncomplicated cases, its applicability in complicated appendicitis warrants careful consideration. Conversion to TPLA or open appendectomy(OA) remains a crucial aspect of assessing the feasibility of TULAA in complex scenarios.

Conversion rates in TULAA vary within the literature, especially when considering complicated appendicitis. A meta-analysis including 16 studies reported an overall conversion rate that was similar between TULAA and TPLA (4). However, a single-center study spanning from 2006 to 2016, encompassing 1,275 children and adolescents, found that both the conversion rate and complication rate were significantly higher in complicated appendicitis (13), and another study reported a 3% conversion rate in a cohort of 316 children undergoing TULAA between 2017 and 2022 (14).

Several patient-related factors have been identified as potential contributors to conversion during TULAA for complicated appendicitis. One frequently cited factor is the anatomical location of the appendix (15).

A high Body Mass Index (BMI), dense adhesions between the appendix and surrounding tissues, and severe inflammation were identified as independent risk factors for prolonged operative time during TULAA (16, 17), which may subsequently increase the likelihood of conversion. However, no cases requiring conversion were observed in this study, which may be related to the extremely low proportion of overweight patients among the study subjects.

Surgeon experience plays a critical role in the efficiency and safety of TULAA. Studies indicate that operative time, a key indicator of surgical proficiency, is influenced by the surgeon's level of experience.

While TULAA has emerged as a promising alternative to TPLA in pediatric appendicitis, several limitations in the existing literature warrant further investigation. One of the primary limitations is the relative lack of high-quality evidence, particularly randomized controlled trials (RCTs). To address this, future research should prioritize well-designed, adequately powered RCTs comparing TULAA with TPLA and OA, with clear inclusion/exclusion criteria and standardized surgical protocols. In addition, most of the current studies on transumbilical single-port laparoscopic appendectomy (TULAA) have included cases of uncomplicated appendicitis. Therefore, further research is also needed to explore the safety and efficacy of TULAA in cases of complicated appendicitis.

## 5 Conclusion

For pediatric acute appendicitis, TULAA is a safe and feasible minimally invasive technique, resulting in a significantly reduced hospital stay compared to TPLA despite a slightly delayed return of bowel function. Both techniques demonstrate comparable operative times and overall complication rates. TULAA represents a valuable surgical option within the pediatric minimally invasive arsenal.

## Data Availability

The raw data supporting the conclusions of this article will be made available by the authors, without undue reservation.
